# Emotional Speech Processing at the Intersection of Prosody and Semantics

**DOI:** 10.1371/journal.pone.0047279

**Published:** 2012-10-31

**Authors:** Rachel Schwartz, Marc D. Pell

**Affiliations:** McGill University, Montréal, Québec, Canada; University Of Cambridge, United Kingdom

## Abstract

The ability to accurately perceive emotions is crucial for effective social interaction. Many questions remain regarding how different sources of emotional cues in speech (e.g., prosody, semantic information) are processed during emotional communication. Using a cross-modal emotional priming paradigm (Facial affect decision task), we compared the relative contributions of processing utterances with single-channel (prosody-only) versus multi-channel (prosody and semantic) cues on the perception of happy, sad, and angry emotional expressions. Our data show that emotional speech cues produce robust congruency effects on decisions about an emotionally related face target, although no processing advantage occurred when prime stimuli contained multi-channel as opposed to single-channel speech cues. Our data suggest that utterances with prosodic cues alone and utterances with combined prosody and semantic cues both activate knowledge that leads to emotional congruency (priming) effects, but that the convergence of these two information sources does not always heighten access to this knowledge during emotional speech processing.

## Introduction

In pop culture, it is often said that it matters less “what” is said than “how” one says it. Is there truth to this adage? How important are the words compared to the tone (i.e., *prosody*) in the perception of emotions in speech, and does this processing differ depending on the emotion expressed? Of special interest here, how does emotional processing change over the course of a spoken utterance that contains vocal cues about emotion *and* an emerging semantic context for interpreting the speaker's emotion state? Is emotion recognition bolstered in some way at the intersection of prosody and semantic cues during emotional speech processing? The current study seeks to address these questions and provide insight on how emotion is implicitly recognized, distinguished, and processed according to the availability of particular cues in speech.

The communication of emotion is dynamic and occurs through multiple channels, including visual facial cues [1]–[3], tone of voice or “emotional prosody” [4]–[7], a speaker's choice of words, i.e. “semantics” [8], or even gesture and body language [9]–[11]. The ability to accurately perceive emotion is crucial for effective social interaction, and requires the integration of multiple cues at a speed that allows for a coordinated exchange with one's conversational partner. In dyadic conversation, the synchronization of prosodic characteristics such as speech rate, vocal intensity, pause patterns, and utterance duration is a noted phenomenon that is thought to facilitate empathy and emotional engagement [12]–[15]. However, many questions remain regarding the manner in which different emotional cues are processed and integrated to infer a speaker's emotional state as humans converse; in particular, it is poorly understood how prosodic cues, which are omnipresent in speech, are modulated by an emerging semantic context conveying emotion as spoken language unfolds and is assigned meaning.

One situation in which the distinction between prosodic and semantic cues is crucial to understanding a speaker's intended meaning is in the communication of irony, and specifically sarcasm. Sarcasm is a type of irony which relies upon the discordance between the literal (semantic) and the nonverbal (prosodic) cues to indicate that the speaker's intended meaning runs counter to the literal meaning [16]. Several recent studies have focused on the relationship between prosodic and textual cues in ironic language [17]–[19], highlighting the importance of exploring incongruencies between channels in order to understand how meaning formulation occurs in spoken communication. While ironic language is not within the scope of the present experiment, the current study will seek to disentangle the relative contributions of prosodic and semantic cues to better determine how emotion is perceived in real-world language processing.

Researchers have studied emotional speech recognition and its processing structure in multiple ways; some have employed explicit tasks of emotion recognition using a gating paradigm (more commonly employed in lexical tasks) to learn at what point in a sentence isolated vocal information allows for recognition of discrete emotion meanings [20]. Others have carefully controlled the duration of vocal exposure and tested either how well subjects were able to explicitly identify the emotion at a given time length [21], or measured emotion activation implicitly through event related potentials [22]–[24]. Still others have attempted to index the amount of time needed for implicit processing of emotional vocal cues by using a priming paradigm to examine which duration of vocal stimuli provided optimal priming, thereby reflecting the time course required for perception of the emotion [21], [25]–[27]. These studies imply that the implicit recognition of discrete emotions occurs very quickly, in the 300–400 ms time window [23]–[25], whereas the ability to *explicitly* name the emotion conveyed by a speaker takes somewhat longer (requiring approximately 500–800 ms of stimulus exposure for most emotional expressions [20]). However, as many studies have presented emotionally-inflected pseudo-utterances or semantically-neutral utterances as their stimuli in order to focus on effects due to emotional prosody (independent of semantics), the interplay of prosody and semantic cues on emotion recognition during on-line speech processing is still coarsely defined (cf. [28]).

Determining the isolated versus combined effects of processing prosody and semantic features is an empirical challenge, as these channels are intricately linked in natural speech. While pseudo-utterances have proven useful in determining the relative hierarchy of prosody and semantics [22], [23], [29] and prosody and situational context [30], these stimuli are quite removed from what would be experienced in the real world and are likely to engender additional cognitive processing demands [27], [31]. Recently, Pell et al. [27] examined the relationship between how prosody-only, semantic-only, or combined prosodic and semantic cues facilitate emotion processing in speech. Their materials consisted of pseudo-utterances for the prosody-only condition, emotional sentences produced with neutral intonation for the semantic-only condition, and emotional sentences produced with a corresponding emotional intonation for the combined prosody-semantic condition. The extent to which different sets of cues implicitly activated discrete emotional meanings in each speech processing condition was evaluated using a priming paradigm (facial affect decision task, (FADT) [32]), where the vocal stimuli always served as primes for a decision about a face target depicting an emotionally congruent, incongruent, or non-emotional (‘grimace’) expression (see [27], [32] for methodological details).

Their data showed that all three sets of cues (prosody-only, semantic-only, and combined prosody and semantic cues) prime decisions about facial expressions that are congruent with the meaning conveyed by speech, although there was no significant difference in the *magnitude* of priming according to which cues were available to the listener. Given the body of research that speaks to there being a processing advantage, in terms of both speed and accuracy, when there is more than one mode or channel of information available [29], [33]–[35], it was somewhat surprising that the combined prosody-semantic condition in Pell et al. [27] did not facilitate emotional processing of the face target to a greater extent. We would have expected to see faster and more accurate responses when both prosody and semantic cues were present, in comparison to the two single-channel conditions, however this did not prove to be the case. The authors postulated that since priming of the face target was always measured from the offset of the utterance prime—for example, at the end of “*They spread false rumors about me”* in the combined prosody and semantic condition—that any facilitative effect of an emerging semantic context when combined with prosodic cues may have dissipated in memory prior to the end of the stimulus prime, yielding the results obtained. The goal of the current investigation was to re-examine the relative priming produced by isolated prosodic cues when compared to combined prosody and semantic cues at the point where the emotional semantic meaning *first unfolds* during on-line speech processing, using similar methods adopted by Pell et al. [27].

Specifically, the present study examined the extent of cross-modal priming (emotional congruency effects) between an utterance prime and an emotional face target in two conditions within the same utterance: a “pre-semantic” condition, where semantic information about emotions was insufficient and only prosodic information signaled discrete emotional meanings; and a “post-semantic” condition, which indexed the point in the utterance where both prosody and semantic cues unambiguously marked the speaker's emotional meaning. This design allowed us to compare any difference in the magnitude of priming associated with the presence of combined channels of emotional speech information in a more sensitive manner than previous undertakings. While conclusions arrived at by Pell et al. [27] were based on happy, sad, and neutral stimuli, the current study included an additional emotional category, anger, to allow for a broader exploration of the differences in temporal and perceptual processes associated with various emotions. Previous literature has established that not all emotions are recognized equally well from either prosody or semantic cues [29]; as a result, we decided to focus on these three well-studied emotions. Based on previous data that speak to a multi-channel advantage during emotion processing (e.g., [33], [35], [36]), we expected to observe congruency effects (i.e., emotion-based priming) in both of our experimental conditions, but greater facilitation of behavioral responses (increased accuracy, faster response times) in the “post-semantic” condition that indexed the convergence of prosody and semantic cues in spoken utterances. We also expected emotion-specific differences in how emotional face targets are processed, such as a processing advantage for happy faces, as has been reported in previous experiments [21], [27], [32], [37].

## Methods

### Ethics statement

This study was ethically approved by the McGill Faculty of Medicine Institutional Review Board in accordance with principles expressed in the Declaration of Helsinki. Informed written consent was obtained for each participant prior to their involvement in the research.

### Participants

Forty volunteers (20 female, 20 male) were recruited through an electronic campus advertisement. Participants averaged 22.5 years of age (*SD* = 2.75) and had completed an average of 17 years of formal education (*SD* = 2.28). All participants had learned English from birth and reported normal (or corrected-to-normal) vision and no hearing difficulties. Subjects were financially compensated for their time.

### Stimuli

The experiment presented truncated recordings of emotionally-inflected English sentences as primes, and facial expressions of emotion as the target stimuli. All utterances and facial stimuli were chosen from perceptually validated datasets and have been used in previous work (e.g., [27], [38]). Many of the utterance primes selected for this study were also presented in the “Prosody-Semantic” condition described by Pell et al. [27], although sentences included here were further manipulated for the onset of semantic cues about emotion, as described below.

a) Primes – The prime stimuli were short (5–8 syllable) English sentences containing emotional prosody that corresponded to the semantics of the sentence, such as “*I was just offered the job*,” spoken in a happy tone of voice. As described in detail by Pell et al. [38], all items were originally produced by two male and two female speakers (lay actors) to convey seven different emotional meanings, with distinct items constructed for each emotional context (approximately 30 sentences per emotion). The intended emotional meaning of each utterance was then verified by a group of 24 listeners who judged “the emotion conveyed by the speaker” in a forced-choice response paradigm. These perceptual measures gathered by Pell et al. [38] were used as a starting point to select potential items for the current study. However, since earlier perceptual judgments of these stimuli were always made after presentation of the full utterance, these measures were insufficient for our present purposes as they do not indicate the extent to which listeners recognized emotions based on prosodic versus semantic information, nor did they inform us of the *onset* of emotional-semantic cues in these items.

To control for these factors, especially the point within the sentence at which an emotion could first be discerned from semantic cues only, a new pilot study using a visual word gating paradigm and the written sentences from Pell et al. [38] was therefore run. Sentences conveying “pleasant surprise” constructed by Pell et al. [38] were omitted as relatively few of these items were correctly identified in the original study. Sixteen participants (undergraduate students at McGill) were presented each sentence word-by-word on a computer screen without any accompanying sound; with the addition of each successive word, subjects were required to categorize the emotional meaning of the sentence as “angry”, “sad”, “happy”, “disgusted”, “fearful”, or “neutral”. Table 1 illustrates the procedure of the pilot study with sample sentences used in the experiment. Subjects were instructed to mark “neutral” if the word(s) did not convey one of the five emotions. The first word in each sentence where recognition of the emotional meaning exceeded 75% correct target responses in the six forced-choice task (where chance = 17%) was designated the emotional “semantic onset” of the sentence. Here, the semantic onset can be thought of as the earliest position within the sentence at which an emotional meaning could be determined in the absence of prosodic cues.

**Table 1:** Examples of the pilot study gating task.

(1) Participant Sees:	(2) Participant Marks	(3) Then Sees:	(4) Marks:	(5) Then Sees:	(6) Marks:	Semantic Onset:
They	NEUTRAL	They spread	NEUTRAL	They spread false	ANGRY	“false”
The	NEUTRAL	The children	NEUTRAL	The children suffered	SAD	“suffered”
I	NEUTRAL	I really	NEUTRAL	I really love	HAPPY	“love”

Based on the new data referring to the semantic word onsets, 40 sentences were selected for presentation in the current experiment: ten “angry”, ten “happy”, ten “sad” and ten “neutral” sentences. In addition to having high recognition rates with respect to the word in the sentence that contained the semantic onset of emotion (approximately 4.5 times chance performance in the pilot study), this set of items controlled for the intra-sentential position of the semantic onset across emotion conditions: 7/10 items per emotion category had a semantic onset in the middle of the sentence (on the third or fourth word) and the remaining three items per category had a semantic onset on the sentence-final word. To control for the position of the semantic onset across emotion conditions, recognition of the semantic word onset was slightly lower (between 56–75% correct) for 5/40 items, although these items were distributed among the three emotion categories (angry, happy, sad). Once specific sentences were selected based on information about their semantic onset, the original pre-recorded versions of these sentences which contained corresponding prosodic cues, produced by one male and one female speaker (from the Pell et al. [38] inventory), served as the actual emotional primes in the experiment. When matching the sentences to the actual recordings, five sentences in each emotional category were arbitrarily assigned to the female voice and the other five to the male voice. Emotion recognition rates for the 40 selected vocal prime stimuli, based on the 24 English participants tested by Pell et al. [38] who performed a seven choice identification task after hearing the full recorded utterances, averaged above 93% per emotion category when participants heard *both* prosody and semantic cues: anger *M = *98%, *SD* = 5; happiness *M* = 93%, *SD* = 8; sadness *M = *96%, *SD* = 6; neutral *M* = 93%, *SD* = 8.

b) Targets - The visual target stimuli consisted of color photographs of facial expressions posed by ten different actors (five male, five female). Each actor contributed six unique expressions: one representing each of the three emotions of interest (anger, sadness, happiness) and three facial “grimaces” that are known not to convey discrete emotional meanings (e.g., [32], [39]). Grimace stimuli, like pseudo-words presented in the lexical decision task, were essential to allow participants to judge the representational status of emotional face targets, or to render a ‘facial affect decision’ that indexes semantic-level emotional processing [25]. A total of 60 unique face targets were presented in different combinations with utterance primes. Recognition rates for the facial stimuli were gathered from 27 subjects in an eight choice identification task, including an open response category. The selected facial stimuli were all correctly identified at rates greater than 94% (with angry faces *M = *94%, *SD = *7; happy faces *M = *99%, *SD = *2; sad faces *M = *97%, *SD* = 4).

### Experimental design

Experimental trials were constructed by pairing individual utterance primes with individual face targets, where sentences were always matched with facial expressions posed by a member of the same sex (although there was no consistent match between the identity of the speaker and the identity of the facial expression throughout the experiment). As in previous administrations of the FADT, half of the trials ended in an emotional (i.e., angry, happy, or sad) face (“YES” trials) and half ended in a grimace face (“NO” trials). For YES trials, the prime-target relationship could be defined in one of three ways: congruent, incongruent, or neutral. Congruent trials were pairings in which both the prime and the target expressed the same emotion (angry-angry, happy-happy, or sad-sad, 30 trials total). Incongruent trials consisted of an angry, happy, or sad emotional utterance matched with a face target displaying each of the two conflicting emotions (e.g., an angry sentence followed by a happy or sad face, 60 trials total). Neutral trials consisted of a neutral sentence followed by an exemplar of each of the three emotional faces (angry, sad, or happy, 30 trials total). An equal number of “NO” trials (*n* = 120) containing emotional or neutral primes matched with a facial grimace target were constructed by pairing each of the 40 utterance primes with three distinct exemplars of a facial grimace. This resulted in 240 unique prime-target combinations in total.

**Figure 1:**
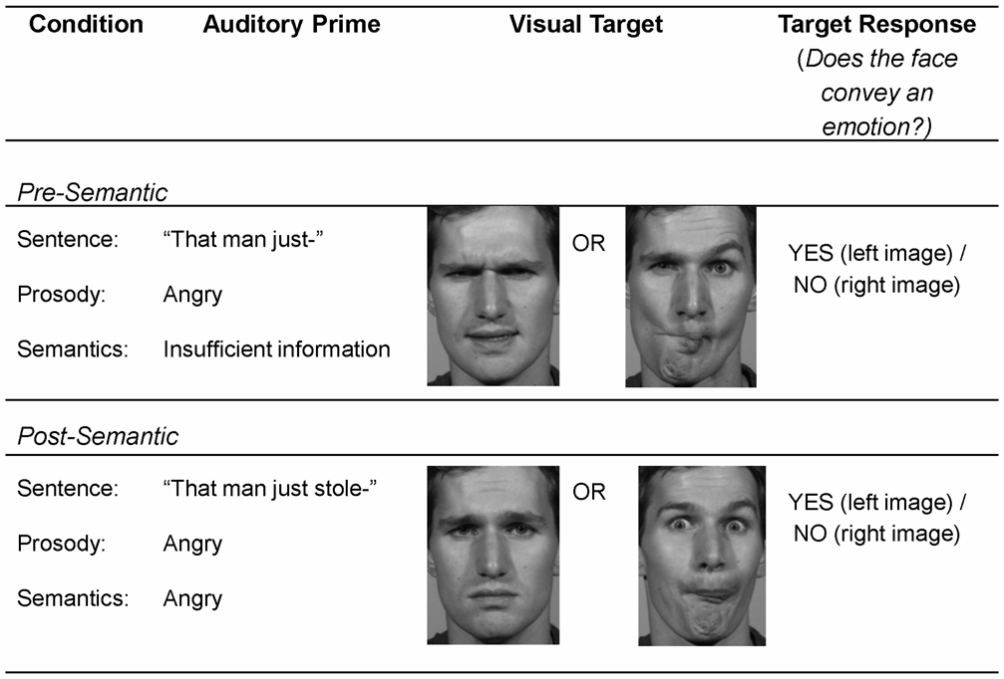
Examples of prime and target stimuli presented in the pre-semantic and post-semantic conditions. For expository purposes, the pre-semantic condition shows a congruent “YES” trial and corresponding “NO” trial, whereas the post-semantic condition shows an incongruent “YES” trial and “NO” trial.

Of major interest here, all prime-target pairings appeared in two separate conditions that differed only in what type of speech cues served to prime emotional processing of the face target: in the “pre-semantic” condition, the prime sentence was truncated at the offset of the word directly preceding the determined semantic onset word; and in the “post-semantic” condition, the sentence was clipped immediately after the semantic onset word. For example, in the sentence, “*That man just stole my wallet*,” separate prime stimuli were constructed around the semantic onset word “*stole*”; in the pre-semantic condition, subjects heard “*That man just—”*, whereas in the post-semantic condition, subjects heard “*That man just stole—“*. In both conditions, the face target appeared at the very offset of the final word of the prime stimulus and participants were required to make a decision about whether or not the face represented an emotion (yes/no response). In the pre-semantic condition (prior to the semantic onset), it is assumed that emotional processing of speech would index only prosodic information, whereas the post-semantic condition would index both prosodic *and* emerging semantic cues representing the speaker's meaning (please see Figure 1 for an illustration of stimuli and the experimental design).

### Procedure

Each participant completed two sessions, each lasting approximately 30 minutes (240 trials×2 conditions). Each session was broken down into six presentation blocks with a rest period between blocks. The post-semantic condition was always presented during the second testing session due to the possible confounds of processing the semantic context of the longer stimuli with priming effects in the pre-semantic condition if the conditions were counter-balanced for presentation order; in order to reduce any potential carryover effects of always completing the pre-semantic condition first, the second testing session always took place at least five days after the first session.

The experiment was controlled using SuperLab 4.0 presentation software (Cedrus Corporation, USA) and all auditory stimuli were presented through headphones. Each trial began with a visual fixation display lasting 350 ms, followed by a 500 ms pause, followed by the utterance prime, and then the presentation of the face target immediately following the offset of the auditory stimulus. Participants were instructed to focus on the face target stimulus to decide whether the facial expression conveyed an emotion by pressing either a YES or NO button on a Cedrus 4-button response box as accurately and quickly as possible. The computer registered the response, the visual stimulus disappeared from the screen, and a 2000 ms inter-trial interval led to the beginning of the next trial. In each condition, each prime sentence occurred six times in distinct prime-target combinations, although there was never a repetition of the same vocal stimulus in any given block. Each presentation block consisted of half grimace (“NO”) trials, two-sixths incongruent trials, and one-sixth congruent trials (relatedness proportion of approximately 17% per block and in the experiment as a whole). Presentation blocks were presented in random order across participants and trials were fully randomized within blocks. Each testing session began with two practice blocks (eight trials each) where participants first learned to judge the emotional status of face targets independent of concurrent primes, followed by the same trials accompanied by auditory primes corresponding to the experimental condition being performed. Participants were paid at the end of the second session.

## Results

The overall error rate for all 40 participants across the Pre- and Post-semantic conditions was 5.4% of all trials (*SD* = 8.3). Based on the overall error patterns, data for one male participant who committed more than 33% errors across YES trials (30% errors in the Pre-semantic condition and 38.3% errors in the Post-semantic condition) were removed from further consideration, as the high error rate suggested that he did not sufficiently understand the task. Unlike previous studies [21], [25], [27], [40] that found higher overall error rates for YES trials when pseudo-utterances were presented, our data show comparable error rates for YES trials (*M* = 5.1%, *SD* = 7.9) and NO trials (*M* = 5.9%, *SD* = 8.9). The low error rates overall demonstrate that participants could reliably differentiate the emotional status of facial expressions (i.e. render facial affect decisions) and complied with task goals throughout the experiment.

To examine priming effects on accuracy and latency measures in each condition, data for YES and NO trials were analyzed separately. Only latencies for trials yielding correct responses were included in the analysis. In order to limit the influence of extreme data points based on the individual response distributions, latency values falling outside of two standard deviations above or below the conditional mean for each participant were normalized by replacing these data points with the corresponding value equal to two standard deviations from the mean in the corresponding direction (5.02% of total values). Accuracy and response times to YES trials were then analyzed with separate 2×3×3 analyses of variance (ANOVAs) with repeated measures on Condition (Pre-semantic, Post-semantic), Face Target (Angry, Happy, Sad) and Voice Prime (Congruent, Incongruent, Neutral). Tukey's HSD post hoc comparisons (*p*<.05) were used to further examine significant main and interaction effects. Table 2 provides a summary of the mean accuracy and latencies for judging face targets preceded by an angry, happy, sad, or neutral prime utterance in the pre-semantic versus post-semantic condition.

**Table 2:** Mean facial affect decision accuracy (% correct) and response latencies (milliseconds) as a function of semantic condition, prime emotion, and face target (standard deviations in parentheses; congruent pairs in bold).

		PRE-Semantic Condition Voice Prime				POST-Semantic Condition Voice Prime			
*Measure*	*Face Target*	*Happy*	*Angry*	*Sad*	*Neutral*	*Happy*	*Angry*	*Sad*	*Neutral*
*Accuracy*	Happy	**98.2**	97.4	95.4	97.7	**99.0**	99.0	97.4	98.2
		**(4.5)**	(5.9)	(7.9)	(7.4)	**(3.1)**	(3.1)	(6.4)	(4.5)
	Angry	90.5	**90.8**	87.4	90.8	91.5	**94.6**	90.8	93.8
		(10.7)	**(9.0)**	(11.6)	(11.1)	(11.1)	**(9.7)**	(10.4)	(10.2)
	Sad	93.8	94.6	**93.6**	95.6	95.1	93.3	**95.9**	96.2
		(8.8)	(7.6)	**(8.7)**	(7.2)	(7.6)	(10.1)	**(6.8)**	(5.9)
	Grimace	92.6	91.0	94.8	93.8	95.4	94.5	95.6	95.2
		(10.1)	(8.6)	(8.7)	(8.4)	(8.2)	(9.8)	(9.4)	(7.7)
*Latency*	Happy	**684**	689	694	694	**637**	661	663	671
		**(120)**	(132)	(133)	(138)	**(99)**	(112)	(113)	(106)
	Angry	817	**801**	853	813	758	**737**	759	753
		(167)	**(177)**	(229)	(151)	(129)	**(130)**	(135)	(121)
	Sad	797	806	**747**	785	752	738	**729**	754
		(162)	(171)	**(146)**	(146)	(140)	(124)	**(152)**	(123)
	Grimace	754	760	772	765	714	711	710	713
		(158)	(188)	(187)	(198)	(134)	(140)	(133)	(132)

### Accuracy

For accuracy, the 2×3×3 ANOVA yielded a main effect of Voice Prime, *F*(2, 76) = 4.01, *p = .*02, η^2 = ^.095, Face Target, *F*(2, 76) = 19.23, *p*<.000001, η^2^ = .336 and Condition, *F*(1, 38) = 7.76, *p*<.01, η^2^  = .170. The main effect of Voice showed that the ability to accurately make emotional decisions about face targets was significantly influenced by the prime-target relationship; faces preceded by a congruent or neutral speech prime promoted more accurate responses when compared to incongruent speech cues overall. Post hoc inspection of the Face main effect revealed that, regardless of prime type or semantic condition, participants responded significantly more accurately to happy faces (98% correct) than to sad faces (95%), which were both judged significantly better than angry faces (92%). Finally, the Condition main effect was explained by the fact that participants made significantly fewer errors in the post-semantic, compared to the pre-semantic, condition (96% vs. 94% accuracy, respectively). There were no significant interactions between Face Target, Voice Prime, and/or Condition for this analysis (Condition×Face interaction: *F* (2,76) = 1.58, *p = *.21; Condition×Voice: *F* (2,76) = .65, *p = *.52; Face×Voice: *F* (4,152) = 1.07, *p = *.37; Condition×Face×Voice: *F* (4,152) = .61, *p = *.65, all interactions *ns*).

Although the primary focus of our study was priming effects observed for the YES trials, we also analyzed data from the NO trials which portrayed facial grimaces. A 2×4 ANOVA was conducted with repeated measures on Condition (Pre-semantic, Post-semantic) and Voice Prime (Angry, Happy, Sad, Neutral). This resulted in significant main effects of Condition (*F*(1, 38) = 8.54, *p*<.01, η^2 = ^.1834) and Voice Prime (*F*(3, 114) = 5.18, *p*<.01, η^2 = ^.1199), as well as a significant interaction of these factors (*F*(3, 114) = 2.89, *p*<.05, η^2 = ^.0706). Post hoc analysis of the interaction showed that this was largely due to the Pre-semantic angry voice primes, which resulted in significantly less accurate “NO” judgments (91%) than in any of the other Pre- or Post-semantic Voice prime conditions. Additionally, facial affect decisions following happy primes were significantly less accurate in the Pre-semantic versus Post-semantic condition.

### Latencies

**Figure 2:**
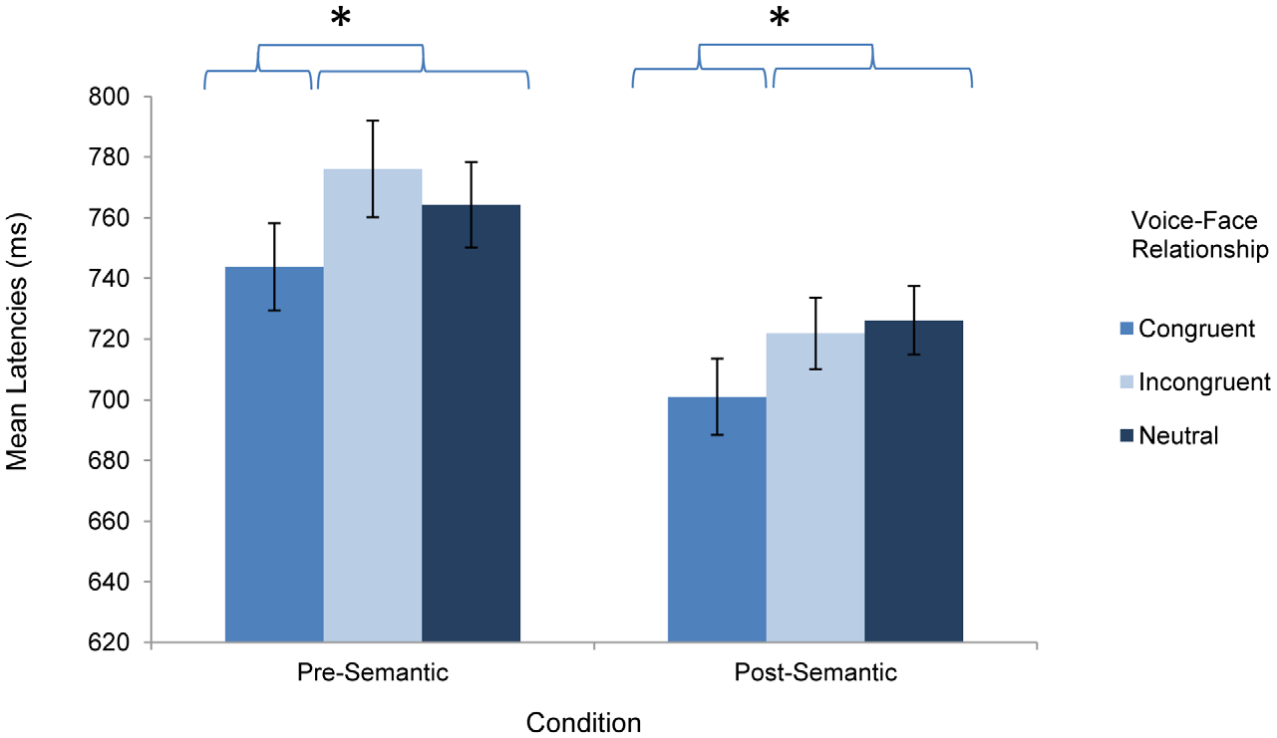
Effects of prime type on response latencies. Effects of congruent, incongruent, and neutral voice primes on facial affect decision latencies (in milliseconds) made immediately prior to the emotional semantic word onset (pre-semantic condition) and immediately following the emotional semantic word (post-semantic condition). Error bars display the standard error of the means.

To test priming effects on response times, a 2×3×3 (Condition×Face Target×Voice Prime) ANOVA performed on correct YES trials produced a significant main effect for Face Target (*F*(2, 76) = 49.72, *p*<.0001, η^2 = ^.5668), Voice Prime (*F*(2, 76) = 23.55, *p*<.0001, η^2 = ^.3826), and Condition (*F*(1, 38) = 10.41, *p*<.01, η^2 = ^.2150). Post hoc Tukey's tests (*p*<.05) on the Face Target main effect showed that participants responded significantly faster overall to happy faces (*M* = 673 ms) than to angry (*M* = 783 ms) or sad faces (*M* = 760 ms), which did not differ significantly. The Voice Prime main effect revealed that participants responded significantly faster overall when speech cues were congruent with the face (*M* = 722 ms) than emotionally incongruent (*M* = 749 ms) or neutral (*M* = 745 ms). In general, response times were significantly longer in the Pre-semantic (*M* = 761 ms) relative to the Post-semantic Condition (*M* = 716 ms). In addition, the Condition×Face interaction was significant (*F*(2,76) = 4.32, *p*<.05, η^2 = ^.1022); this effect simply reinforced that facial affect decisions were rendered more quickly for all emotions in the Post- versus Pre-Semantic condition, with slight variations in the relative pattern of response times to Angry versus Sad faces (which differed significantly only in the Pre-semantic condition). The other two-way interactions were not significant (Condition×Voice Prime: *F*(2,76) = 2.30, *p* = .11; Face×Voice Prime: *F*(4,152) = 1.51, *p* = .20), although the three-way interaction of Condition, Face Target, and Voice Prime represented a trend in the data (*F*(4, 152) = 2.16, *p* = .08). In terms of understanding the influence of voice primes by condition, the three-way interaction was principally explained by the fact that for happy faces, voice-face priming was only significant in the post-semantic versus the pre-semantic condition (all other patterns could be understood from the main effects described above). Figure 2 illustrates the pattern of response times across emotions in the two experimental conditions.

Finally, a 2×4 (Pre-/Post-Semantic Condition×Vocal Prime) ANOVA was run on the latencies for correct NO trial responses. This analysis revealed a significant main effect of Condition (*F*(1, 38) = 10.36, *p*<.01, *η^2^* = .2142) but no significant main or interactive effect involving Vocal Prime. As was seen for YES trials, participants judged face targets significantly slower overall in the Pre-semantic Condition (*M* = 761 ms) when compared to the Post-semantic Condition (*M* = 716 ms).

## Discussion

This study investigated how sad, happy, and angry emotions are encoded over the course of a spoken, meaningful utterance based on the listener's analysis of prosodic and combined prosody and semantic cues. By separating our vocal stimuli into a “Pre-semantic” condition, which contained emotional prosody but no semantic indicators of emotion, and a “Post-semantic” condition, which contained both prosodic and semantic information, we were able to examine the relative contributions of prosody and combined prosody and semantics during on-line processing of emotional speech. Additionally, by including a broader range of emotions than many previous undertakings, our experimental design allowed us to explore whether priming effects and the influence of different cue types generalize across discrete emotion categories.

As has been reported in several studies now [5]–[7], [21], [27], [32], [41]–[44], we observed a robust emotion-congruency effect, characterized by significantly faster and more accurate decisions about an emotional face when the preceding auditory information was emotionally congruent with the target expression. This renewed evidence that emotional cues in speech prime decisions about emotional faces according to the underlying meaning of information in each modality supports the idea that communicative expressions of discrete emotions, such as anger, happiness, and sadness (among others) activate shared knowledge in emotional memory [45], [46]. However, of major interest here, and contrary to our initial hypothesis, our findings suggest that processing prosody-only cues versus combined prosody and semantic cues does not differentially affect the *extent* of priming on the face, or the presumed accessibility or activation level of relevant emotion knowledge, as indicated by the absence of significant interactions between Voice prime and the semantic Condition variables in our measures [27].

While our results appear surprising in the face of literature that speaks to a processing advantage when emotional events are ‘enriched’ or contain multiple cues [7], [47]–[50], this discrepancy may be partly due to methodological differences among studies. As noted by de Gelder & Bertelson [51] and Paulmann & Pell [29], experiments that report a multi-modal processing advantage, and that specifically argue that semantic cues are preferentially attended to over prosody, often lack ecological validity due to the use of pseudo-utterances [22], interference paradigms [45], [49], [52]–[54], or forced-choice conflict situations [22], [28], [55], all of which seldom occur in real-world processing of emotion. Based on our results and those reported by Pell et al. [27], it seems that emotional meanings registered by speech prosody and by combined prosodic and semantic cues hold similar potential to influence related processing in the visual modality in terms of behavioral priming, even when these effects are measured at an emerging semantic context for understanding the speaker's emotion. At the same time, our data did reveal a trend for priming to be greater in the post-semantic condition (although only for happy stimuli), suggesting that further studies will be needed to firmly establish the effects of multi-modal stimuli on emotional memory when prosody and semantic cues converge.

It is also possible that during on-line speech processing, the additive effects of processing both prosody and semantic information occur very quickly and can only be detected using highly sensitive temporal measures such as event-related brain potentials [36]. Schirmer & Kotz [56] proposed a multi-step model for emotional speech processing in which initial perception and acoustic analysis of emotional speech cues take place as early as 100 ms after stimulus onset, and there is evidence that the emotional salience of speech cues and their discrete emotional meanings are registered after 200 ms and approximately 300–400 ms, respectively [23]. Perhaps, as the multi-step process of emotional speech processing is explored further, it will be necessary to combine behavioral and neuroimaging approaches to identify the point at which prosodic and semantic information are first integrated and to elucidate their combined effects on a semantic network for understanding emotions. A more precise knowledge of the time course associated with the integration of different types of speech cues would allow us to gain a better understanding of the role each source of information plays as we interpret real-world social interactions.

However, it may be the case that there is little or no multi-channel advantage for processing combined prosodic and semantic cues in emotional speech [27]. If this is the case, new questions would arise about the process by which we integrate linguistic and non-verbal vocal cues and the underlying neural structures involved. Laurienti, Kraft, Maldjian, Burdette, and Wallace [57] conducted a study that explored whether multisensory (visual and auditory) stimuli would enhance behavioral performance. While they found a definite advantage for cross-modal (visual and auditory) stimuli, no advantage was seen for cues within one domain (such as being presented with a color word simultaneously with the color itself). Could it be that prosodic and semantic cues conveying emotion are sometimes so intricately linked in speech that they function as if within one domain? On a structural level, there does appear to be a very close relationship between semantic and prosodic systems for speech comprehension, such that when meaningless sentences containing only prosodic information are heard, there is a paradoxical increase in semantic processing areas [58], [59]. Interestingly, a study by Regenbogen and colleagues [60] found that replacing an emotional video clip's semantic content with neutral (but meaningful) words decreased activation in semantic-related areas of the brain, although there was a simultaneous increase in activation in auditory and visual processing areas. This finding could speak to the importance our brain places on attending to more naturalistic, meaningful emotional information; indeed, in a similar study by Regenbogen et al. [61] which employed the same stimuli as [60] but focused on physiological measures, the authors concluded that the insertion of neutral semantics was unlikely to reflect real-world speech processing due to the unnatural mismatch between what was spoken and the other two channels (facial and prosodic) for which emotion was present. De Gelder & Bertelson [51], in a review article focusing on studies which sought to replicate real-world emotion processing, noted that stimuli in one modality (either auditory or visual) can activate brain regions associated with the other, non-present modality; this suggests that the lack of a clear multi-channel advantage in our experiment may be due to the fact that certain naturalistic types of single-channel cues are sufficient to activate parts of the brain associated with the processing of multi-channel stimuli [62]. Future studies investigating the activation of brain regions associated with processing different sources of naturalistic emotional speech cues will be beneficial to understanding the neurocognitive operations that underlie the rapid analysis and integration of emotional cues in speech.

One potential concern about the current experiment is that the increased semantic and syntactic content presented in the Post-semantic Condition relative to the Pre-semantic Condition may have influenced our results, particularly response times, in a broader manner. It could be argued that presenting utterances that contain an unequal amount of semantic and syntactic content in one condition relative to the other is unbalanced, as it is clear that semantic and syntactic variations differentially affect cognitive processing demands (e.g. [63]). While our experiment relied upon two conditions which differed in the amount of linguistic content present to disentangle the relative contributions of prosodic and combined prosodic-semantic cues, future studies could usefully eliminate any potential confound increased linguistic content may introduce by developing an experimental paradigm which maintains an equivalent amount of linguistic content across experimental conditions. For example, the prosody-only condition could be constructed so that it is identical to the prosody-semantic condition in linguistic structure, with the only exception being that the prosody-only stimuli would finish with an emotionally-neutral word, while the combined condition would contain semantically-emotional cues. In the case of the current study, however, please note that the effects of increased linguistic content found in our Post-semantic Condition relative to the Pre-semantic Condition were uniformly distributed across emotional categories and prime-target pairings, and therefore would not have detracted from our ability to discern or explain priming effects in our data.

Finally, our new experiment provides further insight on emotional processing beyond the issue of how prosody versus combined prosody and semantic cues are treated by listeners. First, as commonly reported in previous studies using the FADT and elsewhere in the literature, we found an overall performance advantage (less errors, shorter latencies) when participants judged happy face targets when compared to other emotional faces independent of prime type or condition; this result is probably due to the “happy face advantage” that is routinely reported in studies of both explicit and implicit emotion processing of faces (see [27], [64], [65] for further discussion). Another important finding was that overall error and latency rates across emotions were much lower in this study than in previous FADT priming studies that presented pseudo-utterances as primes [21], [27], [32], [40]. It has been suggested that pseudo-utterances tend to increase cognitive processing demands and promote interference (e.g., slower or more error-prone responses) in behavioral tasks that have used these stimuli in order to effectively isolate prosody from semantics [27], [47]. This possibility seems likely in light of functional imaging studies which have revealed greater activation of the left perisylvian semantic areas during processing of unintelligible speech than during processing of normal speech [58], [59]. Given our finding that *absolute* measures of accuracy improved and response times to judge emotional faces were shorter when regular English utterances versus pseudo-utterances were presented as primes, one can argue that processing pseudo-utterance stimuli has a general cost on the cognitive processing system, which interferes with concurrent judgments (such as facial affect decisions), although emotional cues in both types of stimuli hold equal potential to produce priming in an emotion-congruent manner [21], [27], [32].

In conclusion, the present study extends the results of Pell et al. [27] by demonstrating that priming is not systematically enhanced through the presence of multi-channel speech cues in a carefully controlled design that identified the onset of semantically congruent information about emotions in the utterance. Our data imply that prosodic cues alone, when naturalistic and unambiguous, are sufficient to effectively register and interpret emotions in spoken language and that this effect persists at the intersection of prosody and semantic cues in speech. Future studies will undoubtedly shed further light on how and when prosodic and semantic sources of information are combined in natural communication and how they interact in an ongoing manner as listeners process speech.
